# Spatial and Temporal Clustering of Dengue Virus Transmission in Thai Villages

**DOI:** 10.1371/journal.pmed.0050205

**Published:** 2008-11-04

**Authors:** Mammen P Mammen, Chusak Pimgate, Constantianus J. M Koenraadt, Alan L Rothman, Jared Aldstadt, Ananda Nisalak, Richard G Jarman, James W Jones, Anon Srikiatkhachorn, Charity Ann Ypil-Butac, Arthur Getis, Suwich Thammapalo, Amy C Morrison, Daniel H Libraty, Sharone Green, Thomas W Scott

**Affiliations:** 1 Department of Virology, Armed Forces Research Institute of Medical Sciences (AFRIMS), Bangkok, Thailand; 2 Department of Entomology, University of California, Davis, California, United States of America; 3 Center for Infectious Disease and Vaccine Research, University of Massachusetts Medical School, Worcester, Massachusetts, United States of America; 4 Department of Geography, San Diego State University, San Diego, California, United States of America; 5 Department of Entomology, AFRIMS, Bangkok, Thailand; 6 Office of Disease Prevention and Control, Ministry of Public Health, Nonthaburi, Thailand; Hong Kong University, Hong Kong

## Abstract

**Background:**

Transmission of dengue viruses (DENV), the leading cause of arboviral disease worldwide, is known to vary through time and space, likely owing to a combination of factors related to the human host, virus, mosquito vector, and environment. An improved understanding of variation in transmission patterns is fundamental to conducting surveillance and implementing disease prevention strategies. To test the hypothesis that DENV transmission is spatially and temporally focal, we compared geographic and temporal characteristics within Thai villages where DENV are and are not being actively transmitted.

**Methods and Findings:**

Cluster investigations were conducted within 100 m of homes where febrile index children with (positive clusters) and without (negative clusters) acute dengue lived during two seasons of peak DENV transmission. Data on human infection and mosquito infection/density were examined to precisely (1) define the spatial and temporal dimensions of DENV transmission, (2) correlate these factors with variation in DENV transmission, and (3) determine the burden of inapparent and symptomatic infections. Among 556 village children enrolled as neighbors of 12 dengue-positive and 22 dengue-negative index cases, all 27 DENV infections (4.9% of enrollees) occurred in positive clusters (*p* < 0.01; attributable risk [AR] = 10.4 per 100; 95% confidence interval 1–19.8 per 100]. In positive clusters, 12.4% of enrollees became infected in a 15-d period and DENV infections were aggregated centrally near homes of index cases. As only 1 of 217 pairs of serologic specimens tested in positive clusters revealed a recent DENV infection that occurred prior to cluster initiation, we attribute the observed DENV transmission subsequent to cluster investigation to recent DENV transmission activity. Of the 1,022 female adult *Ae. aegypti* collected, all eight (0.8%) dengue-infected mosquitoes came from houses in positive clusters; none from control clusters or schools. Distinguishing features between positive and negative clusters were greater availability of piped water in negative clusters (*p* < 0.01) and greater number of *Ae. aegypti* pupae per person in positive clusters (*p* = 0.04). During primarily DENV-4 transmission seasons, the ratio of inapparent to symptomatic infections was nearly 1:1 among child enrollees. Study limitations included inability to sample all children and mosquitoes within each cluster and our reliance on serologic rather than virologic evidence of interval infections in enrollees given restrictions on the frequency of blood collections in children.

**Conclusions:**

Our data reveal the remarkably focal nature of DENV transmission within a hyperendemic rural area of Thailand. These data suggest that active school-based dengue case detection prompting local spraying could contain recent virus introductions and reduce the longitudinal risk of virus spread within rural areas. Our results should prompt future cluster studies to explore how host immune and behavioral aspects may impact DENV transmission and prevention strategies. Cluster methodology could serve as a useful research tool for investigation of other temporally and spatially clustered infectious diseases.

## Abstract 2

Investigating dengue cases identified by testing febrile schoolchildren in rural Thai villages, Mammen P. Mammen and colleagues find a pattern of focal spread to houses neighboring those of case patients.

## Editors' Summary

**Background.:**

Every year, over 50 million people living in tropical and subtropical urban and semi-urban areas become infected with dengue (a mosquito-borne viral infection) and several hundred thousand develop a potentially lethal complication called dengue hemorrhagic fever. Dengue is caused by four closely related viruses that are transmitted to people through the bites of infected female Aedes aegypti mosquitoes. These day-biting insects, which breed in household water containers and in the water that collects in used tires and other discarded containers, acquire dengue virus through feeding on the blood of an infected person. Some people who become infected with dengue virus have no symptoms but others develop high fever, a rash, and severe headache that lasts two to seven days. In dengue hemorrhagic fever, small blood vessels become leaky, which causes nose and gum bleeds, bruising and, in the worst cases, failure of the circulatory system and death. There is no specific treatment for dengue fever or dengue hemorrhagic fever but standard medical care—in particular, replacement of lost blood fluids—helps most people survive the latter condition.

**Why Was This Study Done?:**

There is no vaccine to prevent dengue. As a result the only way to minimize dengue outbreaks is to control mosquito numbers through environmental management—providing piped water, encouraging people not to store water in open containers, and removing other sources of standing water—and by applying insecticides to areas where mosquitoes breed. During outbreaks, because *Ae. aegypti* mosquitoes rest in houses, insecticides are also often sprayed in dwellings in the affected areas. However, to improve dengue prevention and surveillance, public-health officials need to know much more about the patterns of dengue virus transmission and about the factors that underlie these patterns. In this study, therefore, the researchers test the idea that dengue virus transmission occurs in localized neighborhood clusters over short periods of time.

**What Did the Researchers Do and Find?:**

The researchers used “cluster investigations” to examine the pattern of dengue virus transmission among school children in several rural villages in Thailand, a country where dengue is very common (hyperendemic). Primary school children with fever were identified during two seasons of peak dengue virus transmission. Each child was characterized as a dengue-positive index case (by finding dengue virus in their blood) or as a dengue-negative index case. Data on human infection and mosquito infection and density were then collected within 100 meters of the homes of each index case—the “cluster area.” Not all the neighbors of the index cases participated in the study but among the 556 village children who did participate, there were 27 dengue infections, all of which occurred in clusters centered on the homes of the dengue-positive index cases. In the positive clusters, one in eight of the enrolled children became infected within 15 days of the index case becoming ill. Among 1,000 *Ae. aegypti* mosquitoes collected inside and around the houses in each cluster, only eight were infected with dengue and these were all collected from houses in positive clusters. Finally, there was a greater availability of piped water and fewer *Ae. aegypti* pupae in the negative clusters than in the positive clusters.

**What Do These Findings Mean?:**

Although this study did not sample all the children or mosquitoes within each cluster area, these findings show that in an area where dengue is hyperendemic, dengue virus transmission among children occurs in localized areas and over short time periods. The findings also suggest that focal transmission is associated with recent dengue virus introductions and that one or a few mosquitoes are likely responsible for all the transmission in each cluster. Although it would be impractical to set up surveillance of all the school children in Thailand for dengue infections, these findings suggest that improved detection of cases within schools combined with local spraying inside the homes in the immediate vicinity of any affected children could help to halt dengue virus transmission. Future cluster studies could explore how human behavior and human immunity affect dengue virus transmission and could also be used to investigate other temporally and spatially clustered infectious diseases, including malaria.

**Additional Information.:**

Please access these Web sites via the online version of this summary at http://dx.doi.org/10.1371/journal.pmed.0050205.

- Read the related *PLoS Medicine* Perspective by Steven Riley
- The US Centers for Disease Control and Prevention provides detailed information about dengue fever, including a questions and answers section in English and Spanish
- The World Health Organization provides information on dengue and dengue hemorrhagic fever around the world (in several languages)
- Links to additional information about dengue are provided by MedlinePlus (in English and Spanish)

## Introduction

Dengue is the leading cause of human arboviral disease worldwide. Dengue viruses (DENV) of the family *Flaviviridae* and genus *Flavivirus*, co-circulate as four antigenically related serotypes (DENV-1, −2, −3, and −4), each in varying annual frequencies in Thailand [1] and other tropical countries. The container-breeding mosquito Aedes aegypti (L.) serves as the primary vector responsible for DENV transmission within human populations. Females feed preferentially and frequently on human blood and consequently live in and around human dwellings [2,3]. Transmission of DENV to humans results in either inapparent infection, undifferentiated febrile illness, dengue fever (DF), or life-threatening dengue hemorrhagic fever (DHF). Except for a few notable exceptions, vector control (larvicide treatments, insecticide sprays, and source reduction) has been ineffectively implemented, and no vaccine or clinical cure is yet available for use. Consequently, DENV remain a major cause of morbidity in the tropics and threaten to further expand geographically.

DENV transmission and disease are determined by a combination of factors [4] involving the human host [5–7], virus [8–11], mosquito vector [12,13], and environment [13]. Although past studies have revealed general temporal and spatial patterns in the distribution and abundance of *Ae. aegypti* and human DENV infections [14–18], greater resolution of transmission dynamics across finer geographic and temporal scales is needed to refine current dengue surveillance and control strategies.

In an earlier prospective cohort study of schoolchildren in Thailand, Endy and others [19] reported a nonuniform distribution of DENV illness and viral serotypes. To test the hypothesis that DENV transmission is spatially and temporally focal, we extended the school-based study design to include cluster investigations [20] in villages associated with schools. By sampling children and mosquitoes within the neighborhood of children absent from school with fever and dengue viremia, we hypothesized that we would be able to detect, in the same general area and time, other human and mosquito infections and more precisely identify determinants of transmission risk. We used school-based dengue cases to trigger village surveillance of children and mosquitoes within spatial and temporal clusters. We sought a rigorous study of cluster areas over a 15-d period to more accurately define the burden of DENV within a prescribed area (both inapparent and symptomatic infections) and its relationship to mosquito density and infectivity. On the basis of our data, we aimed to consider implications on improving disease prevention strategies.

## Methods

### Study Area and Selection of Schools and Villages

**Figure 1:**
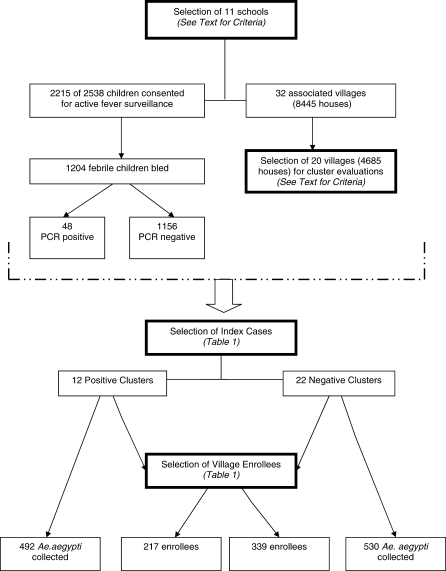
Study Design Overview

Our study area (Muang District, Kamphaeng Phet Province [KPP], north-central Thailand [19]) is, by Thai standards, relatively sparsely populated with 233,033 residents in 63,500 houses in an area encompassing 1,962 km^2^. The average temperature is 28.0 °C with an average monthly rainfall of approximately 200 mm during the rainy months of May to October (National Statistical Office). We selected 11 participating primary schools on the basis of higher numbers of hospitalized dengue cases amongst their students during the prior 5 y, proximity to our field station, and interest of the school administrators. Selected schools (Figure 1) were associated with 32 villages (8,445 houses). Given the workload limitations of entomological surveys, 20 of these villages (4,685 houses) were selected for inclusion on the basis of the density of houses, favoring those with houses in close proximity of each other (<100 m). We assigned unique house codes to these 4,685 houses and entered the associated spatial coordinates and the total number and demographics of residents into a Geographic Information Systems (GIS) [15] database (MapInfo [2000] version 6.0; MapInfo Corporation).

### Selection of School Children for Initiation of Village Clusters

Following parental written consent, 2,215 children were enrolled in a school cohort and were followed using an active school-absence–based surveillance for febrile illnesses [19]. From this cohort, 1,204 children with febrile illnesses were evaluated between June and November of 2004 and 2005. Those meeting eligibility criteria (Table 1) were considered “index” cases to initiate clusters, though not necessarily implying that they were newly introducing the virus into the study area. Positive clusters were triggered by index cases with laboratory-confirmed dengue viremia determined within 24 h of fever presentation; negative clusters by a febrile child without dengue viremia (and subsequent evidence of lack of DENV seroconversion). On only one occasion, a viremic index case could not be subsequently confirmed as having an acute DENV infection by seroconversion and was reclassified as triggering a dengue-negative cluster. Homes of index cases served as the center-points for clusters. Following written parental consent, we enrolled ten to 25 children (6 mo to 15 y of age) residing within a 100-m radius (reflecting the flight range of female *Ae. aegypti* [21]) of the index case's home. Because of the workload necessary to conduct each cluster investigation, we studied no more than three clusters in a single week, precluding our conducting cluster investigations for all index cases that were otherwise suitable. When possible, we studied a pair (positive and negative) of nonoverlapping clusters, spatially matched (index cases within a 5-km distance) and temporally matched (initiation of both clusters within 5 d of each other).

**Table 1:**
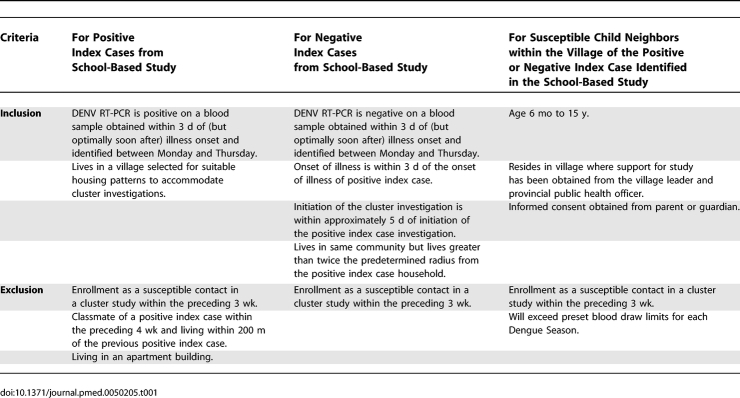
Eligibility Criteria for Enrollment in Cluster Investigations

### Clinical Evaluations of Village Enrollees

On days 0, 5, 10, and 15, village enrollees were administered an oral thermometer (axillary, if age <2 y) and questionnaires documenting symptoms experienced over the preceding 5 d. Blood was drawn on days 0 and 15; aliquots were barcoded to assure technician blinding. DENV infections were identified by a dengue/Japanese encephalitis IgM/IgG enzyme-linked immunosorbent assay (ELISA), conducted by the Armed Forces Research Institute of Medical Sciences (AFRIMS) Bangkok facility, which permits the identification of “acute” dengue cases (with further classification into primary or secondary) as well as “recent” dengue cases occurring up to 60 d prior [22,23]. Dengue viremia and serotype were determined by reverse transcriptase-polymerase chain reaction (RT-PCR)/nested PCR (referred to here as PCR) conducted at the KPP-AFRIMS Virology Research Unit (KAVRU) on day 0 and 15 sera. Village enrollees were classified as dengue-positive based on ELISA seroconversion between acute and convalescent sera. If a convalescent specimen was not available, then a positive PCR, either on days 0 or 15, was considered as indicative of a dengue-positive case only if the PCR could be confirmed in the AFRIMS Bangkok laboratory using a different aliquot of the same serum. If any day 15 sera were PCR positive, those enrollees were further evaluated by thermometer and questionnaire on days 20, 25, and 30 but without drawing a convalescent serum to assess for ELISA seroconversion. Dengue cases were classified as inapparent (lack of subjective and objective fever, defined as ≥38 °C) or symptomatic dengue. Symptomatic dengue was further classified as nonhospitalized dengue (nhD), hospitalized DF (hDF), and hospitalized DHF (hDHF) as previously described [19]. Grading of DHF cases followed World Health Organization guidelines [24].

### Entomologic Evaluations

All entomologic field personnel were blinded to the PCR result of the index case. On day 1, female adult *Ae. aegypti* were collected using backpack aspirators from inside and immediately surrounding each home within the cluster [12]. *Ae. aegypti* larvae and pupae were collected and source containers characterized (using the SUM method) as previously described [17,25,26]. Mosquitoes were collected from the school classroom and bathroom of the index case on the same day. Measures of adult and immature mosquito abundance were used to estimate entomological risk, including the House Index (percentage of houses infested with *Ae. aegypti* larvae and/or pupae), Container Index (percentage of water-holding containers infested with *Ae. aegypti* immatures), Breteau Index (number of *Ae. aegypti*-positive containers per 100 houses), and pupal and adult *Ae. aegypti* densities.

### Entomologic Interventions

After mosquitoes were collected, a pyrethrin mixture (BP-300: Pyronyl oil concentrate OR-3610A, Prentiss Inc.) was sprayed to kill adult mosquitoes inside and immediately surrounding each home [27]. Temephos (Abate) was applied to water-holding containers to kill larvae. Our intent was to terminate DENV transmission at the start of each cluster so that any dengue cases subsequently identified reflected transmission that occurred prior to cluster initiation. As the Thai Ministry of Public Health (MoPH) policy requires insecticide spraying both upon identification of a dengue case and 7 d later, the study team provided the initial spraying and the Thai MoPH provided the day 7 spraying (deltamethrin or permethrin 10%), regardless of the dengue PCR status of the index case for that cluster.

### Laboratory Preparation of Collected Mosquitoes

Female *Ae. aegypti* were screened for DENV by RT-PCR using a modified protocol [28]. Briefly, pools of ten mosquitoes were made by combining 14 μl from individual mosquito suspensions (in 100 μl of RPMI containing 1% L-glutamine and 10% heat-inactivated FBS) and clarified by centrifugation at 8,000 rpm at 4 °C for 20 min. From positive pools, individual mosquitoes were assayed by serotype-specific PCR using 14 μl of the original suspension in 126 μl of diluent.

### RT-PCR Assay on Human Sera and Mosquito Suspensions

Virus RNA was extracted from 140 μl of human serum or mosquito suspension using Qiagen Viral RNA Extraction kits. Serotype-specific DNA fragments from each unknown sample were amplified by TaqDNA polymerase through RT-PCR performed at KAVRU following modifications to the Lanciotti protocol [29].

### Statistical Methodology for Data Analysis

Data were analyzed using STATA (Stata Statistical Software, version 7) and SPSS (SPSS for Windows, version 15) software. We analyzed demographic characteristics of enrollees and environmental attributes at the cluster and house levels. In view of the cluster design, we calculated the incidence of dengue by taking mean cluster level event rates. We then computed the attributable risk (AR) as the difference between the incidence rates in positive and negative clusters. To compare positive and negative clusters, we employed an independent *t*-test weighted by the number of houses per cluster. The null hypothesis that there was no relationship between the likelihood of DENV infection and distance to the index case was evaluated using Fisher's exact test. Five mutually exclusive residential distance categories were used in this analysis (0–20 m, >20–40 m, >40–60 m, >60–80 m, and >80–100 m). In order to evaluate for a distance effect in conjunction with enrollee demographics, a multivariate logistic regression model was formulated.

### Scientific and Ethical Review and Approval

The study protocol and consent forms were approved by the AFRIMS Scientific Review Committee and the ethical review committees of the U.S. Army Surgeon General, Thai MoPH, University of California at Davis, University of Massachusetts Medical School, and San Diego State University.

## Results

### Initiation of Cluster Investigations

Of the 1,204 febrile children (506 in 2004 and 698 in 2005) who provided blood specimens, 48 (28 in 2004 and 20 in 2005) had detectable DENV viremia. Thirty-four cluster investigations were conducted during the study period (Table 2). Ten clusters (five pairs) in 2004 and two clusters (one pair) in 2005 were spatially and temporally matched. The sex and age distribution of the positive and negative index cases were similar. Children in 58% (seven of 12) of the positive clusters (six in 2004 and one in 2005) attended a single school (school number 2).

**Table 2:**
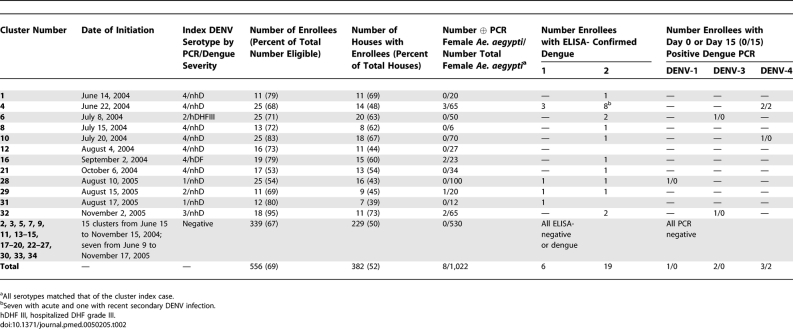
Summary of Cluster Investigations

### Cluster Enrollees

**Figure 2:**
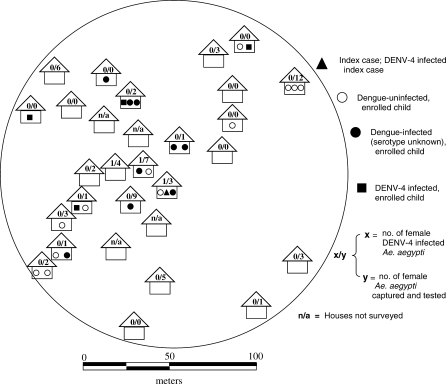
Intense DENV Transmission in Cluster 4 Cluster number 4 illustrates extensive DENV transmission occurring within a 15-d period. In comparison, the paired negative cluster (cluster number 5, not shown) included 22 houses, 21 *Ae. aegypti*, and 15 contacts with no evidence of DENV transmission within a 15-d period. These index cases were 258 m apart and the cluster investigations were initiated 2 d apart.

**Figure 3:**
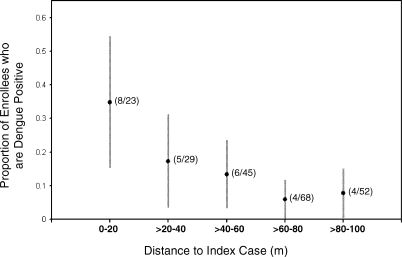
Clustering of DENV Infections within Positive Clusters This graph shows the relationship of distance between the houses of enrollees and the index case in the positive clusters and the proportion of those enrollees that experienced DENV seroconversion. Error bars represent 95% CIs of the proportions. Numbers in parenthesis indicate the number of positive enrollees and the total number of enrollees in each distance interval. The relationship between distance and the proportion of enrollees that are dengue positive was significant (Fisher's exact test, *p* < 0.001).

Among the 556 village enrollees (217 in positive and 339 in negative clusters), 27 DENV infections were detected during the 15-d follow-up period. These incident infections occurred exclusively in positive clusters (*t*-test; *p* < 0.01; AR = 10.4 per 100; 95% confidence interval [CI] 1–19.8 per 100). This result represented a 4.9% risk among enrollees for experiencing a DENV infection within 15 d of cluster initiation, but a 12.4% risk among enrollees who resided in a positive cluster. Cluster number 4 (Figure 2) contributed disproportionately to this difference. However, all but one positive cluster (cluster number 12) exhibited at least one neighbor with dengue within the 15-d period. There was a statistically significant clustering of DENV cases close to the center of positive clusters when we examined all positive clusters together (Figure 3). Demographics of enrollees between positive and negative clusters were comparable (Table 3). There was no difference in distance between the index cases and respective enrollees in the positive and the negative clusters.

**Table 3:**
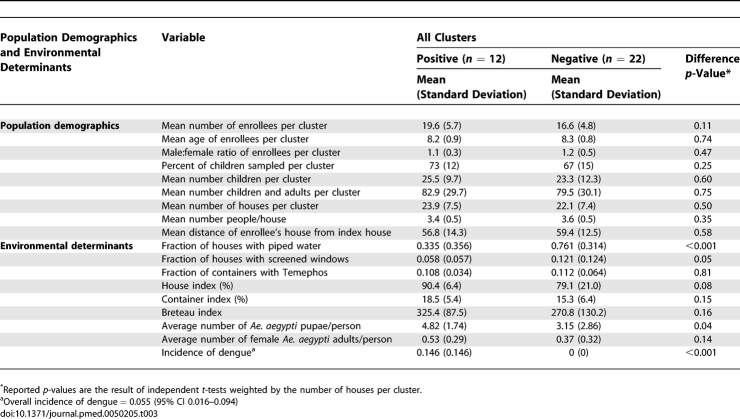
Comparison of Dengue-Positive and Dengue-Negative Clusters

A multivariate logistic regression model was estimated to examine the focal nature of transmission while controlling for cluster demographics. Distance between the house of each enrollee and the index case was the measure of focality. An indicator variable was used to account for the evidently excessive transmission in cluster number 4. The model included the age and gender of the enrollees as well as the interaction of these two variables. Resulting coefficient estimates, standard errors, and *p*-values are given in Table 4. A diagnostic test does not indicate a lack of fit (Hosmer-Lemeshow test, *p* = 0.23) [30]. A negative and significant parameter estimate indicated that the probability of infection decreased as the distance between enrollees and the index house increased. Modeling results also indicate a gender difference in the effect of age on the probability of infection. The probability that a male enrollee seroconverted decreased with age. This effect was not observed among female enrollees, in whom older enrollees had a higher probability of infection. These trends are apparent in the distribution of infections (Figure S1; Table 5).

**Table 4:**
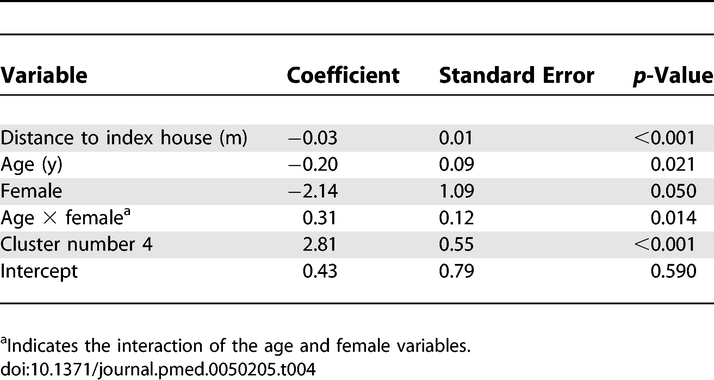
Results of Multivariate Logistic Regression Analysis

**Table 5:**
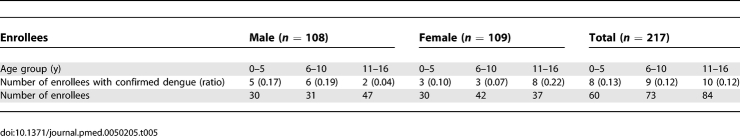
Infections among Enrollees in Positive Clusters by Gender and Age Group

Clustering was additionally observed within households as has been previously described [31]. Relative risk of dengue seroconversion among household enrollees of a dengue versus non-dengue case was 2.63 (95% CI 0.96–7.21; Pearson's Chi^2^ test) with an absolute risk of 6.88 per 100 (95% CI 0–17.29), indicating a strong, but not statistically significant trend towards household risk.

Of the 27 DENV infections among village enrollees (Table 6), 14 were inapparent, and 13 were symptomatic. Inapparent infections were more likely with primary (five out of six) than secondary (seven out of 19) DENV infections (*p* = 0.05; Pearson's Chi^2^ test). All but one positive cluster (cluster number 6) had concordance of serotypes between the index case and viremic enrollees. (Pearson's Chi^2^ test used.)

**Table 6:**
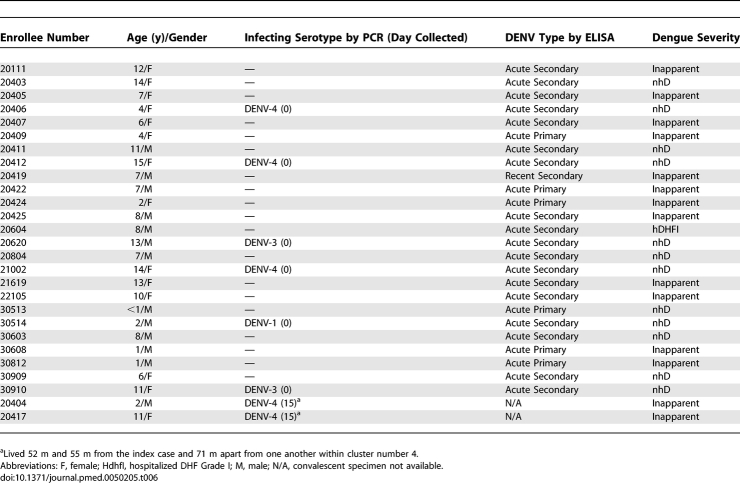
Clinical Spectrum of Illness among 27 Enrollees with DENV Infections

### Environmental Determinants of Transmission

Among environmental features evaluated **(** Table 3), positive clusters were less likely to have piped water than were negative clusters**.** Though the number of water-holding containers was similar in houses with and without piped water (17.6 ± 8.6 versus 17.8 ± 8.1, *t*-test, *p* = 0.28), containers with *Ae. aegypti* larvae or pupae were significantly less abundant in houses with than without piped water (3.2 ± 3.0 versus 4.4 ± 3.3, *t*-test, *p* < 0.001). Use of the larvicide Temephos was higher in the schools than in the villages; 43% and 30% of containers had Temephos in schools in 2004 and 2005, respectively. On average 10% of containers had Temephos in the villages during both study years.

### Mosquito Collections and Spraying

A total of 1,022 adult female *Ae. aegypti* were collected from within and immediately surrounding homes (Figure 1; Table 2) of which eight (0.8%) were PCR-positive. The average proportion of houses sampled was 0.92 in the positive clusters and 0.93 in the negative clusters (*t*-test, *p* = 0.53). Average number of *Ae. aegypti* pupae/person was significantly higher in positive clusters (Table 3)**.** Although no significant differences were detected, all classical entomological indices (House, Container, and Breteau) and average number of female *Ae. aegypti* adults/person were higher in positive clusters. The average proportion of houses sprayed was 0.87 in the positive clusters and 0.84 in the negative clusters (*t*-test, *p* = 0.39). A total of eight female *Ae. aegypti* were collected from schools associated with cluster initiation; none were PCR-positive.

## Discussion

Although focal DENV transmission has been noted previously [14,15,32], to our knowledge this is the first study to demonstrate, using control clusters and precise human and entomological data, recent DENV transmission that was focal through space and over a short time span (15 d). DENV-infected hosts (27 enrollees) and vectors (eight *Ae. aegypti*) were exclusively identified in the 12 dengue-positive clusters, despite a nearly 1:2 ratio of enrollees between positive and negative clusters. Furthermore, we observed significant central clustering of DENV cases within positive clusters.

We suspect that focal transmission was associated with recent DENV introductions because of the 217 paired serologic specimens from positive cluster enrollees, only one revealed an elevated but declining immunoglobulin M level, which would be indicative of a recent DENV infection occurring up to 60 d prior to cluster initiation [22]. Consequently, we attributed the observed DENV transmission (enrollees with viremia on day 0 or 15 and/or seroconversion between days 0 and 15) to recent virus introductions. This conclusion is in contrast, however, to data published by Beckett and others [20] who conducted cluster investigations in West Jakarta, Indonesia. They detected 175 recent DENV infections upon enrollment in 53 positive clusters compared to our one in 12 positive clusters, arguing against recent virus introduction. We attribute these contrasting results to study design differences. First, we recruited from schools whereas Beckett recruited from a hospital, potentially after the virus had undergone significant community-based amplification. Second, we preferentially enrolled children as the primary susceptible and amplifying portion of the host population. Beckett additionally enrolled adults. Adults may have exhibited greater background dengue immunity that may have confounded the serologic data. Third, Beckett's study was conducted in an urban area, in contrast to rural villages in our study. Differences in transmission dynamics between these kinds of habitats were likely shaped by the frequency of DENV introductions and diversity in human behaviors.

Previous studies have documented hyperendemicity of all four DENV serotypes with an approximate 5% annual risk of acquiring an infection in KPP [19]. In our study, cluster number 4 had a 52% attack rate among enrollees sampled during the 15-d follow-up period. However, after excluding this cluster and its matched negative cluster, the adjusted AR remained high (six per 100). This number represented a 12.4% risk of an enrolled child acquiring a DENV infection within a 15-d period when living within 100 m of a child ill with dengue. Eleven of 12 positive clusters had at least one enrollee with acute dengue in addition to the index case. Given the required intrinsic incubation period, and the finding that all eight virus isolates from mosquitoes matched the serotype recovered from the index case suggest, though not definitively, that except for children from whom virus was recovered on day 15, multiple viremic children within a cluster were infected by one or very few infected mosquitoes. Other evidence within our study to further support village- and not school-based vector sources of DENV infection are that: (1) mosquito populations in schools were extremely low, (2) children seroconverting to dengue within a cluster attended different classrooms within the school, (3) genomic sequences of the envelope (E)-regions of the viruses isolated from children and mosquitoes within the same villages were identical (R.G. Jarman, unpublished data), and (4) housemates of dengue seroconverters had a higher relative risk for DENV infection than those of nondengue seroconverters. The latter observation is consistent with previous reports [14–16]. We suspect that the predominance of DENV transmission in KPP villages reflects, at least in part, routine and effective vector control in schools (insecticide every May and July and Temephos to containers every 3 mo), but not in village homes.

Differences in transmission observed between positive and negative clusters could not be attributed to differences in enrollee demographics. Differences in behavioral factors, however, could not be excluded. Within positive clusters, risk of infection decreased with age for males and increased with age for females. This observation merits further investigation with a larger sample and analysis of sex-specific behaviors that might modify risk of infection with advancing age.

The only statistically significant determinant among environmental features associated with focal DENV transmission was the greater availability of piped water in negative clusters. Though one may consider a causal relationship (that is, less piped water availability leading to greater need for water storage leading to more containers for larval mosquito development resulting in higher dengue risk), we found no difference in the number of containers between cluster types. Although accurate data on water turn-over are difficult to obtain, the greater number of positive containers in positive than in negative clusters could not be explained by a difference in the frequency of container turn-over rates that we measured. These data could reflect a historical norm or behavior in response to lack of reliability of piped water possibly guided by people's knowledge of dengue preventive measures [33].

**Figure 4:**
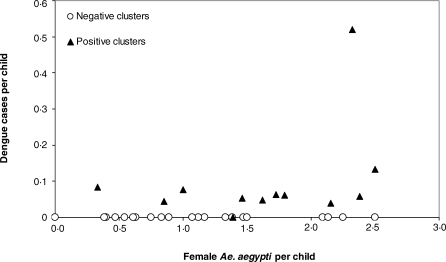
Relationship between Vector Density and Dengue Cases Relationship between the number of *Ae. aegypti* females per child and dengue transmission within 12 positive and 22 negative cluster investigations in 2004 and 2005. Dengue transmission is expressed as the number of positive PCRs on days 0 or 15 of study or of dengue seroconversions between days 0 and 15 per child per cluster.

The only statistically significant difference among entomological indices was the greater number of *Ae. aegypti* pupae per person in positive than negative clusters. It is important to note that observed mean pupae per person exceed by an order of magnitude the minimum entomological threshold estimated by Focks and others [34] for a different region of Thailand. This implies that even when pupal densities are relatively high, differences in this measure of entomological risk can be epidemiologically informative. Although adult mosquito population density tended to be higher in positive clusters, differences were not statistically significant, perhaps due to limitations in sampling adult *Ae. aegypti* with backpack aspirators. Alternatively, mosquito density may be most informative when viewed in concert with herd immunity, and mosquito density alone may be less relevant than the presence of DENV-infected mosquitoes that potentially can transmit virus to multiple individuals [2,3]. Dengue cases in enrollees occurred over a wide range of female *Ae. aegypti* densities (Figure 4). At densities higher than approximately 1.5 *Ae. aegypti* females per child, clusters were more likely to be positive than negative. This indicates that DENV transmission was more likely to occur at higher vector densities.

Perifocal spraying is a common approach by health departments to contain/control dengue. However, this practice has been found to be ineffective in aborting DENV transmission [13,35]. Our data suggest that if school-based surveillance can be bolstered by rapid, easy-to-use, and affordable diagnostics, spatially and temporally focused vector control in rural areas such as KPP could be more effectively applied to contain new virus introductions and offset the theoretical risk of longitudinal transmission within and beyond village foci. Although the risk of infection decreased significantly with distance from the center of a cluster, we did not examine people living beyond 100 m of an index case. Our study did not define the spatial dimensions of DENV transmission. Nevertheless, we expect that interventions will need to go beyond a 100 m radius of the home of a DENV-infected child because viremic residents or visitors bitten by an infected mosquito can move virus farther than a flying, infected adult female *Ae. aegypti* [13,35].

We do not know the longitudinal effects of killing adult mosquitoes on transmission within a community. Koenraadt and others [27] determined in our study area that within 1 wk of spraying insecticide inside homes, approximately 50% of prespraying levels of *Ae. aegypti* populations were reestablished. Identifying only two of 217 child enrollees with dengue viremia on day 15, both approximately 50 m from the index case within the same positive cluster, indicates that vector control can be locally successful when promptly and properly applied in response to a dengue case. Insecticide applications are most effective when applied inside homes where most *Ae. aegypti* rest [12] and otherwise avoid contact with insecticides applied outdoors [35–37].

Though our study design was rigorous, our conclusions must be considered in the context of largely logistical limitations: (1) We did not sample all children and mosquitoes within the cluster area. (2) We were unable to characterize the serotype of all DENV infections among village enrollees given restrictions in the frequency of collecting blood from children. (3) We did not collect data on human mobility/behavior that may have influenced the dynamics of transmission within the villages. (4) The possible contribution of adults to DENV transmission was not studied. (5) We did not study the seroprevalence profiles of cluster enrollees. Future studies should focus on positive clusters to more fully characterize the transmission dynamics, the impact of human behavior on transmission patterns, the appropriate spatial scale for disease surveillance/control, and identify more practical and cost-effective approaches to rapid dengue diagnosis.

Our cluster methodology provided additional epidemiologic insights. Of note, 14 of the 27 cases of dengue among enrollees were clinically inapparent during this period when DENV-4 was the primary serotype circulating. Most (five of six) primary DENV infections detected in our study were clinically inapparent, similar to observations during a predominantly DENV-2 transmission year in Bangkok [38]. The nearly 1:1 ratio of inapparent to symptomatic secondary DENV infections in our study is also consistent with previous results from KPP [19]. DHF occurred in one (8%) of 12 symptomatic infections and one (4%) of 27 DENV infections confirming that severe dengue represents only a small fraction of the total DENV burden. Future cluster studies can complement these clinical and virologic data by examining correlates of protection that limit transmission, early immunologic events via postinoculation pre-illness specimens and their association with disease severity and sequence variation among viruses through time and space as they circulate between human and mosquito hosts.

The prospective cluster methodology utilized here and by others [20] has the potential for broad application. It can be used for multidisciplinary transmission studies of other vector-borne viral diseases as well as spatially and temporally clustered infectious diseases.

## Supporting Information

Figure S1 The Predicted Probability of Infection for Enrollees within Positive Clusters as a Function of Distance to the Index House The probabilities are given for males and females ages 3, 8, and 13 y. Model parameters are reported in Table 5.
(51 KB DOC)

## Abbreviations

AFRIMS: Armed Forces Research Institute of Medical Sciences
AR: attributable risk
CI: confidence interval
DENV: dengue viruses
DF: dengue fever
DHF: dengue hemorrhagic fever
hDF: hospitalized DF
hDHF: hospitalized DHF
KAVRU: KPP-AFRIMS Virology Research Unit
KPP: Kamphaeng Phet Province
MoPH: Ministry of Public Health
nhD: nonhospitalized dengue
PCR: reverse transcriptase-polymerase chain reaction
