# Supplementary material for: Spatial and Temporal Clustering of Dengue Virus Transmission in Thai Villages
Source: PLoS Med. 2008 Nov 4;5(11):e205. doi: 10.1371/journal.pmed.0050205 (PMC2577695; doi:10.1371/journal.pmed.0050205)
Supplement: Supplementary file 1 [file pmed.0050205.sg001.doc]

Probability of Infection

Distance to Index House

3 yr. old female

8 yr. old female

13 yr. old female

8 yr. old male

13 yr. old male

3 yr. old male
